# Information-Theoretic Features of Many Fermion Systems: An Exploration Based on Exactly Solvable Models

**DOI:** 10.3390/e23111488

**Published:** 2021-11-10

**Authors:** Angel Ricardo Plastino, Diana Monteoliva, Angelo Plastino

**Affiliations:** 1 CeBio—Departamento de Ciencias Básicas, Universidad Nacional del Noroeste de la Prov. de Buenos Aires, CONICET, Junin 1988, Argentina; 2 UNLP—Comisión de Investigaciones Científicas Provincia de Buenos Aires La Plata, La Plata 1900, Argentina; monteoli@fisica.unlp.edu.ar; 3 Instituto de Física La Plata—CCT-CONICET, Universidad Nacional de La Plata, C.C. 727, La Plata 1900, Argentina; plastino@fisica.unlp.edu.ar

**Keywords:** exactly solvable model, pairing interaction, monopole interaction

## Abstract

Finite quantum many fermion systems are essential for our current understanding of Nature. They are at the core of molecular, atomic, and nuclear physics. In recent years, the application of information and complexity measures to the study of diverse types of many-fermion systems has opened a line of research that elucidates new aspects of the structure and behavior of this class of physical systems. In this work we explore the main features of information and information-based complexity indicators in exactly soluble many-fermion models of the Lipkin kind. Models of this kind have been extremely useful in shedding light on the intricacies of quantum many body physics. Models of the Lipkin kind play, for finite systems, a role similar to the one played by the celebrated Hubbard model of solid state physics. We consider two many fermion systems and show how their differences can be best appreciated by recourse to information theoretic tools. We appeal to information measures as tools to compare the structural details of different fermion systems. We will discover that few fermion systems are endowed by a much larger complexity-degree than many fermion ones. The same happens with the coupling-constants strengths. Complexity augments as they decrease, without reaching zero. Also, the behavior of the two lowest lying energy states are crucial in evaluating the system’s complexity.

## 1. Introduction

The study of finite many-fermion systems has been enriched in recent years with the incorporation of new mathematical tools inspired in information theory. In particular, information measures and information-based complexity measures have been successfully applied to elucidate various aspects of the physics of atoms, molecules, and atomic nuclei [1,2,3,4,5,6,7,8,9,10,11,12,13,14]. Unfortunately, finite many-fermion systems rarely admit exact analytical treatment, and most studies must rest heavily on the numerical solution of the equations describing the system. It is therefore desirable to incorporate exactly soluble models to the ongoing research program of applying information-theoretical tools to finite many-fermion physics. The aim of the present contribution is to apply information techniques to investigate the properties of exactly soluble many-fermion models akin to the celebrated Lipkin one. Models of the Lipkin kind play for finite systems a role similar to that of the celebrated Hubbard model for solid state physics [15]. Having exact solutions at hands helps quite a lot in understanding what is involved in the variegated approximation invented in the many body field so as to perform approximate treatments devised for making realistic situations tractable ones. Information-theoretic treatments of many body behavior at finite temperature have been lately shown to be a source of insights into the many body problem as well [16]. We will apply in this effort to exactly solvable fermion models the relatively new notions of disequilibrium and statistical complexity. SC-like effects were empirically detected long ago in microscopic systems (nuclear physics and metal clusters) [13,17,18,19,20,21,22], and references therein], being also influenced by mean-field and odd-nucleon blocking effects [23,24].

Encouraged by the above results we revisit here a similar but not identical scenario to deal with two different exactly solvable (interacting) finite fermions-model of the Lipkin kind [see, for instance, [25,26] and references therein] that do not appeal to pairing interaction as in [1]. We will show that the different structural details that characterize the two distinct systems can be well described by the canonical ensemble methodology.

The accompanying order- disorder interplay is described via Gibbs’ canonical ensemble considerations [27] in which the concomitant probability distribution is proportional to $\exp\left(-\beta \hat{H}\right)$, with $\hat{H}$ standing for the Hamiltonian and β for the inverse temperature. Maximum disorder is associated to a uniform distribution (UD) in which all micro-states are equiprobable. The distance in probability space between the actual probability distribution and the UD is called the *disequilibriumD* that is a statistical quantifier that increases as order augments. If we multiply *D* with the entropy *S* we obtain the statistical complexity C=SD. We will profusely use *D* and *C* below as quantum statistical quantifiers.

We will appeal to the Lipkin Model (LM) [25,26], that has proved to be very useful in intense research that revolved on the validity and/or usefulness of several theoretical techniques devised for investigating multiple facets of the fermion many body problem. The LM is based on an SU(2) algebra and produces easily accessible exact solutions. Lipkin-like models are arguably the simplest non trivial finite many-fermion systems. They constitute an ideal testing ground for the application of information theoretical methods to many-fermion systems so as to gain insights that the study of other, more realistic models, can not yield. Both, the Lipkin and the AFP models are two-level nuclei, that provide an extremely simplified model of an atomic nuclei. Any nuclei spectrum displays a complex discrete spectrum and a continuous one. The model retains only the two lowest lying levels.

## 2. Lipkin Model

The model [19,25,26] deal with of N=2Ω fermions distributed between (2Ω)-fold degenerate single-particle (sp) levels separated by a sp energy gap ϵ. Two quantum numbers (μ and *p*) are assigned to a general single particle state. The first takes the values μ=−1 (lower level) and μ=+1 (upper level). The *p* quantum number, often denominated quasi-spin or pseudo spin, picks out a specific belonging to the *N*-fold degeneracy. The couple p,μ may be viewed as a ”site” that is occupied or empty. We have

(1) N=2J,

where *J* standing for a kind of angular momentum. In the wake of Lipkin et al. [25] we define the quasi-spin operators

(2) $${\hat{J}}_{+}=\sum_{p}C_{p,+}^{†}C_{p,-},$$

(3) $${\hat{J}}_{-}=\sum_{p}C_{p,-}^{†}C_{p,+},$$

(4) $${\hat{J}}_{z}=\sum_{p,\mu }\mu \;C_{p,\mu }^{†}C_{p,\mu },$$

(5) $${\hat{J}}^{2}={\hat{J}}_{z}^{2}+\frac{1}{2}\;\left({\hat{J}}_{+}{\hat{J}}_{-}+{\hat{J}}_{-}{\hat{J}}_{+}\right),$$

where the eigenvalues of ${\hat{J}}^{2}$ are of the form J(J+1). It is convenient to define now the operators [25,28]

(6) $${\hat{G}}_{ij}=\sum_{p=1}^{2\Omega }\;C_{p,i}^{†}C_{p,j}.$$

The Likpin Hamiltonian reads

(7) $${\hat{H}}_{L}=\epsilon \sum_{i}^{N}{\hat{G}}_{ii}+\left(v/2\right)\sum_{i<j}^{N}\;\left({\hat{G}}_{ij}+{\hat{G}}_{ji}\right).$$

It commutes with all the $\hat{J}$ operators and thus can be easily diagonalized in any ${\hat{J}}^{2}$-multiplet [25,26,28,29]. *v* is the coupling constant of the two-body interacting part of the Hamiltonian. The effects we wish to study here appealing to information theory quantifiers depend basically on the *v*-value, which acts as the control parameter of the system.

Another useful, exactly solvable Hamiltonian that we will use here is the so-called Abecasis-Faesler-Plastino (AFP)-one [28,29] that reads, using *v* as the coupling constant for the two body interaction (control parameter of the system). The great advantage of the AFP model is that its Hamiltonian is analytically diagonalizable (Lipkin’s is not). Remark that the AFP model exhibits a level-crossing in the ground state energy (see [29]). The lowest lying of the Hamiltonian’s eigenvalues is called the ground state level (gsl). As the coupling constant grows, there is a change in which of the Hamiltonian’s eigenvalues becomes the gsl. The value of the coupling constant *v* at which the level-crossing occurs is loosely referred to as a ”critical coupling constant”. There are several of them. The larger *N*, the larger the number of the level crossings [29].

(8) $${\hat{H}}_{AFP}=\epsilon \sum_{i}^{N}{\hat{G}}_{ii}+v\left({\hat{J}}_{x}-{\hat{J}}_{x}^{2}\right).$$

Here ${\hat{J}}_{x}$ is the exceedingly well known linear combination $[{\hat{J}}_{+}+{\hat{J}}_{-}]/2$. ${\hat{H}}_{AFP}$ also commutes, of course, with all the $\hat{J}$ operators. Note that below, whenever we state that *v* is large or small, this is always in relation to the ϵ-value.

## 3. Hamiltonian Matrices

In the case of the Abecasis-Faesler-Plastino (AFP) model we have, from Equation (6) of [29] the Hamiltonian matrix

(9) $$\begin{array}{ccc} \langle n^{\prime }|H_{AFP}|n\rangle & = & \left(n-J\right)\delta_{n^{\prime },n}+\frac{1}{2}v\{2\left(2J^{2}+J+n^{2}-2Jn\right)\delta_{n^{\prime },n}\\ & & +2\sqrt{\left(2J-n)(n+1\right)}\delta_{n^{\prime },n+1}+2\sqrt{(2J-n+1)n}\delta_{n^{\prime },n-1}\\ & & -\sqrt{\left(2J-n-1)(n+2)(2J-n)(n+1\right)}\delta_{n^{\prime },n+2}\\ & & -\sqrt{(2J-n+2)(n-1)(2J-n+1)n}\delta_{n^{\prime },n-2}. \end{array}$$

The corresponding Lipkin matrix is [26]

(10) $$\begin{array}{ccc} \langle n^{\prime }|H_{L}|n\rangle & = & \left\{\{\frac{N}{2}-n+1-\left((Nn-\frac{N}{2}-n^{2}+2n-1)\right)\omega \}\right\}\delta_{n^{\prime },n}-\\ & & -\frac{v}{2}\sqrt{(N-n)(N-n+1)(n+1)n}\;\delta_{n^{\prime },n+2}\\ & & -\frac{v}{2}\sqrt{(N-n)(N-n+1)(n+1)n}\;\delta_{n^{\prime },n-2}, \end{array}$$

with n=0,1,⋯,N for N=2,4,6,⋯ and J=N/2. After numerically diagonalizing the matrices we find energy-eigenvalues $E_{n}\left(v,J\right)$ for both Hamiltonians. With them we can do statistical mechnics calculations in the canonical ensemble.

## 4. Listing the Main Information-Theoretic Thermal Quantifiers

The main thermal quantifiers of any physical system derive from its partition function *Z* [27], that in turn is constructed with the probabilities associated to the pertinent microscopic states of energies $E_{i}$ [27]. We mention here the mean energy *U*, the entropy *S*, and the free energy *F* [27]. The partition function *Z* [27] and its associated quantifiers are based upon the canonical probability distributions [27] $P_{n}\left(v,J\beta \right)$,with ${}^{\prime }beta$ the inverse temperature. As stated above, we speak of the mean energy U(v,J,β), entropy *S*, and free energy *F*. The pertinent expressions read

(11) $$\begin{array}{ccc} P_{n}\left(v,J,\beta \right) & = & \frac{1}{Z(v,J,\beta )}e^{-\beta E_{n}\left(v,J\right)} \end{array}$$

(12) $$\begin{array}{ccc} Z(v,J,\beta ) & = & \sum_{n=0}^{N}e^{-\beta E_{n}\left(v,J\right)}\\ U(v,J,\beta ) & = & \langle E\rangle =-\frac{\partial lnZ(v,J,\beta )}{\partial \beta }=\\ & = & \sum_{n=0}^{N}E_{n}\left(v,J\right)P_{n}\left(v,J,\beta \right)= \end{array}$$

(13) $$\begin{array}{ccc} & = & \frac{1}{Z(v,J,\beta )}\sum_{n=0}^{N}E_{n}\left(v,J\right)e^{-\beta E_{n}\left(v,J\right)} \end{array}$$

(14) $$\begin{array}{ccc} S(v,J,\beta ) & = & -\sum_{n=0}^{N}P_{n}\left(v,J,\beta \right)\ln\left[P_{n}\left(v,J,\beta \right)\right] \end{array}$$

(15) $$\begin{array}{ccc} F(v,J,\beta ) & = & U(v,J,\beta )-T\;S(v,J,\beta ). \end{array}$$

### Complexity Associated Quantum Quantifiers

More sophisticated quantifiers than the above ones were devised around 25 years ago [30,31,32,33,34,35,36] (they use Equations (11), (12), (13), (14), (15). We pass here to them, specialized for the Likpin and AFP models. Remark that they depend upon the coupling constant of the concomitant two body interaction. They are the disequilibria $D_{AFP}$ and $D_{L}$, together with the statistical complexities $C_{L}\left(AFP\right)$ and $C_{L}\left(L\right)$. Calling $P^{u}$ the uniform distribution we have:

(16) $$\begin{array}{ccc} D_{AFP}\left(v,J,\beta \right) & = & \sum_{n=0}^{N}{\left((P_{n}^{AFP}\left(v,J,\beta \right)-P_{n}^{u})\right)}^{2} \end{array}$$

(17) $$\begin{array}{ccc} D_{L}\left(v,J,\beta \right) & = & \sum_{n=0}^{N}{\left((P_{n}^{L}\left(v,J,\beta \right)-P_{n}^{u})\right)}^{2} \end{array}$$

(18) $$\begin{array}{ccc} C_{L}\left(v,J,\beta \right) & = & S(v,J,\beta )D(v,J,\beta ), \end{array}$$

with a similar expression for $C_{AFP}\left(v,J,\beta \right)$. Remembering now that our *J*-multiplets contain 2J+1=N+1 possible micro-states, one has

(19) $$\begin{array}{c} P_{n}^{u}=\frac{1}{N+1}\;\;\;\forall \;n=0,1,\cdots N. \end{array}$$

The disequilibium *D* is a measure of order [30,31,32,33,34,35,36,37], that is larger the greater its numerical value. D=0 entails total disorder (randomization) [30]. The statistical complexity vanishes both for total order and total disorder [30]. It is maximal when the system attains special kinds of states like those corresponding to ”phase transitions”, more properly crossing levels.

We will find below, in our numerical results, that sometimes *D* reaches a minimum for some specific values of the coupling constant *V*. This fact entails that for this *V*-value, the system attains a maximum of structural looseness, that is, a maximum of randomness.

Let us insist: the disequilibium *D* is a measure of order [30,31,32,33,34,35,36,37], that is larger the greater its numerical value. D=0 entails total disorder (randomization) [30]. The statistical complexity vanishes both for total order and total disorder [30]. Its is maximal, as stated above, when there are level-crossings.

## 5. First Results

**Figure 1:**
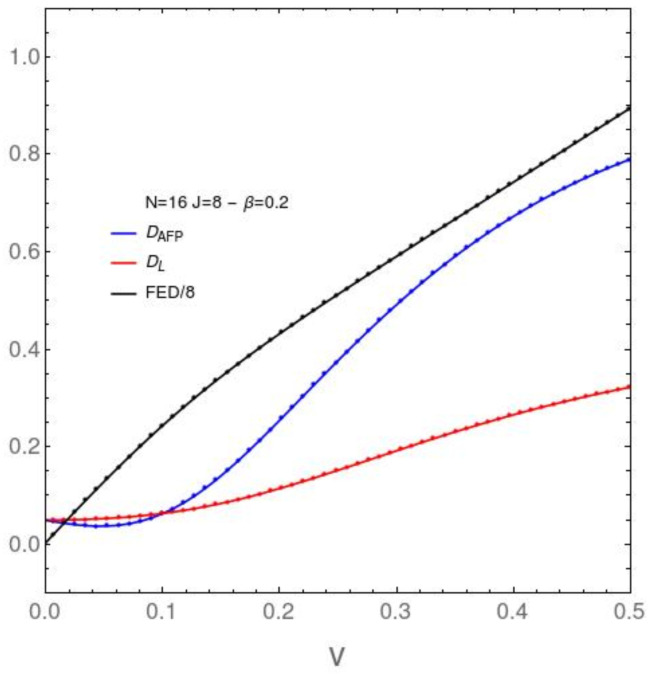
$D_{AFP}$, $D_{L}$, and FED*vs.v*, for N=16 and the relatively high temperature associated with β=0.2. Note that one detects some structural changes only for small *v* (in relation to the ϵ-value).

**Figure 2:**
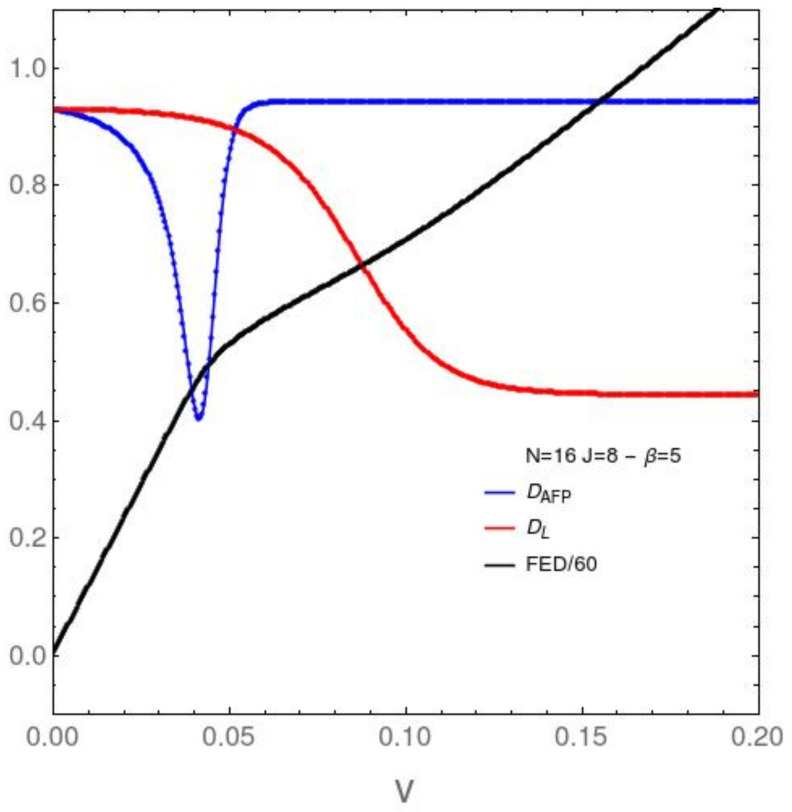
Vertical axis: $D_{AFP}$, $D_{L}$, and FED *vs.* *v* (horizontal axis), for N=16 and the relatively low temperature associated with β=5, Note that one appreciates structural changes only for small *v* (in relation to the ϵ-value.) See text for their meanings.

**Figure 3:**
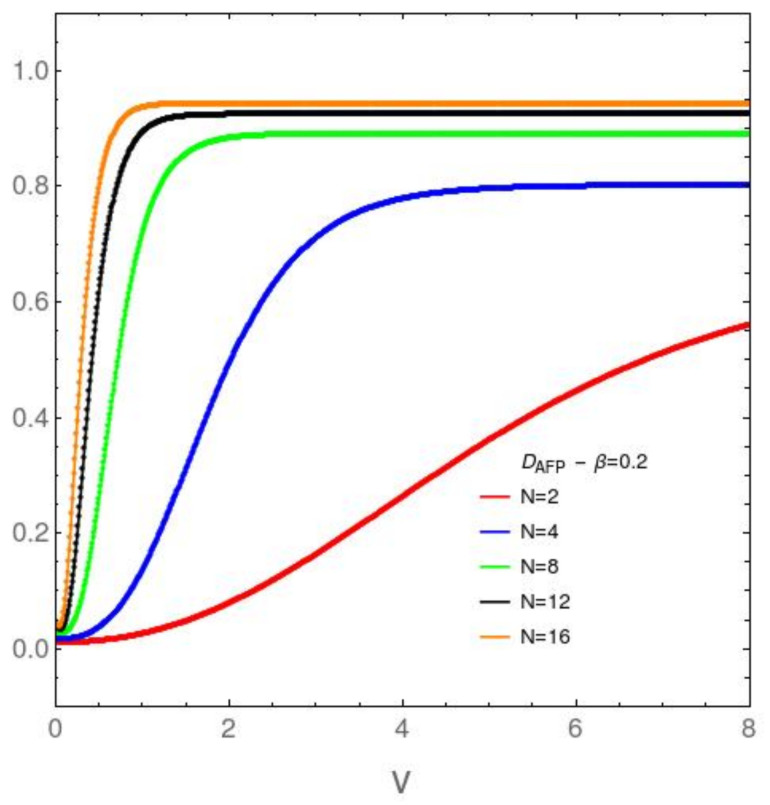
AFP model: $D_{AFP}$ in the vertical axis *vs. v* in the horizontal one, for variegated *N* values and the relatively high temperature associated with β=0.2. As *N* grows, the disequilibrium *D* approaches a step function at the origin, for small *v* in relation to the ϵ-value. Note that only for small *N* one can discern noticeable behavior-change.

We will depict the behavior of our information-theoretic quantifiers [vertical axis] *vs. the interaction strength v* [horizontal axis], with β=0.2 (Figure 1) or β=5.0 (Figure 2), and several values for *N* in Figure 2 and Figure 3. We begin with the disequilibria *D* for our two models ($D_{AFP}$ (blue cirve) and $D_{L}$) (red curve). We plot also the free-energies differences $FED=F_{AFP}-F_{L}$ between them (black curve). The difference between the free energies of two distinct systems (at the same temperature) has been shown to be a good objective quantifier of the degree of distinctness between two systems [38]. We see that it grows uniformly with the interaction strength. For *D*, one detects some structural changes only for small coupling constants.

In our next plot (Figure 2) we go to much lower temperatures and see more interesting effects. The free energy divergence (back curve) exhibits a slope-change at v∼0.5. Near it, the AFP disequilibrium displays a sharp minimum. We associate these to effects for the ground state crossing level (CL) taking place in this *v*-vicinity [29] and correct section below. The unperturbed ground state becomes mixed with existed states at the CL and this mixing generates disorder. At a slightly larger *v* the Lipkin disequilibrium descends, indicating disorder-growth, which in turn entails the mixing just described above. This is an original present finding, because these effects was not known previously.

## 6. Studying the *N* Dependence of Our Quantifiers

**Figure 4:**
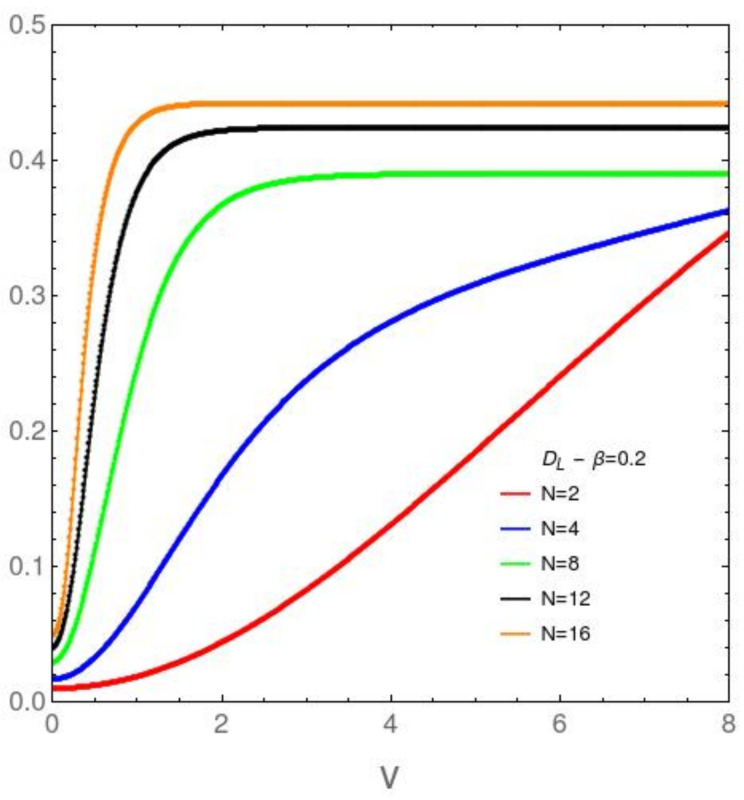
Lipkin model: $D_{L}$ in the vertical axis *vs. v* in the horizontal one, for variegated *N* values and the relatively high temperature associated with β=0.2. As *N* grows, the disequilibrium *D* approaches a step function at the origin, for small *v* in relation to the ϵ-value. Note that only for small *N* one can discern noticeable behavior-change.

**Figure 5:**
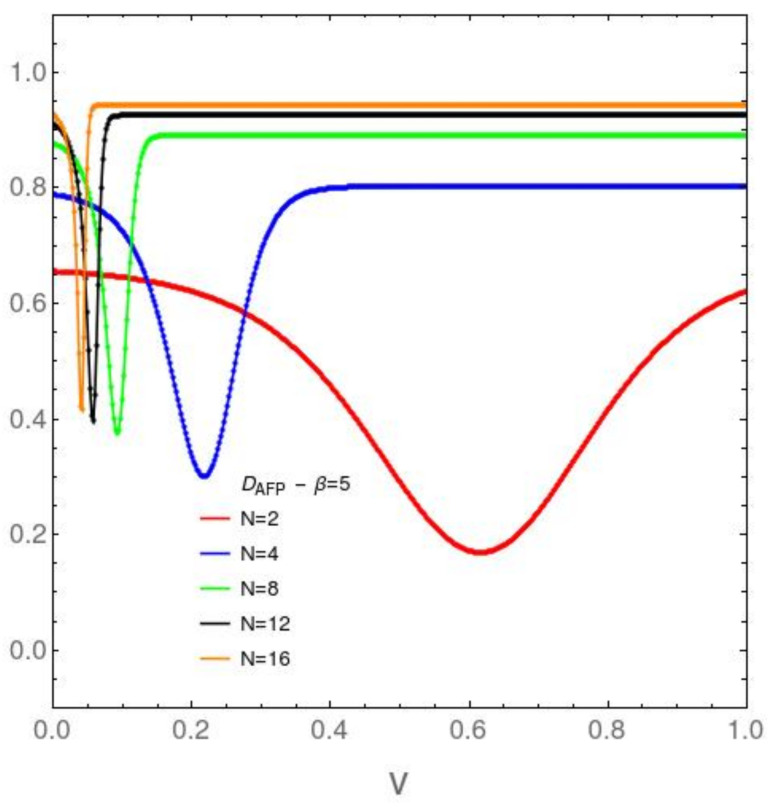
AFP model: $D_{AFP}$ in the vertical axis *vs. v* in the horizontal one, for variegated *N* values and the relatively low temperature associated with β=5. *D* displays a minimum, an interesting feature. The minimum moves towards the origin as *N* grows. After this minimum is reached, for larger coupling constants *D* grows and stabilizes itself near its maximum possible value of unity, indicative of large structural order. Again, interesting physics is detected at small *v* or *N*.

**Figure 6:**
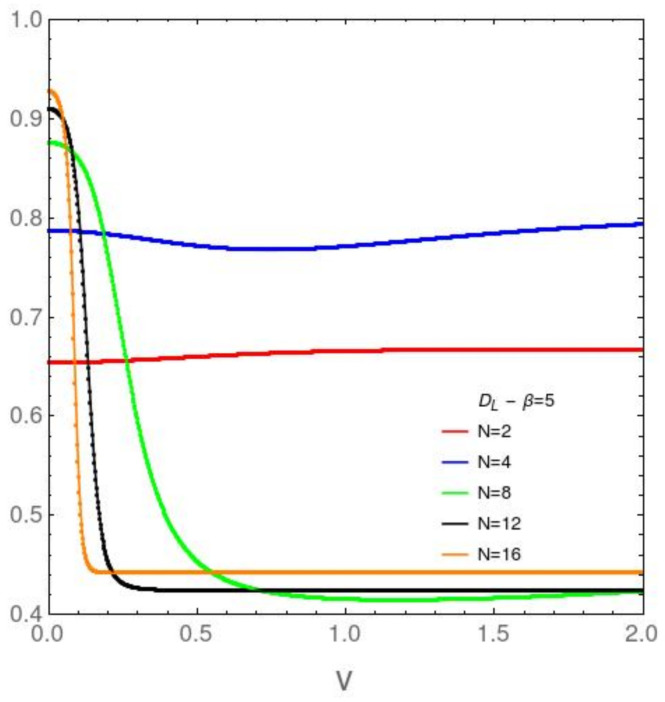
Lipkin model: $D_{L}$ in the vertical axis *vs. v* in the horizontal one, for variegated *N* values and the relatively low temperature associated with β=5. The behavior is quite different from that of the AFP model. An enormous difference is detected between low and large *N* curves. Starting at N=6, *D* decreases (as *v* grows) towards a stable small value, indicative of low structural degree.

It We focus attention now upon the *N* dependence of the disequilibrium and the free energy divergence FED between our two models in Figure 3, Figure 4, Figure 5 and Figure 6. Figure 3 refers to the AFP model for β=0.2, while Figure 4 is devoted with the same temprature. Figure 4 and Figure 5 are like Figure 3 and Figure 4, but at the lower temperature β=5. It is clear that *D* growths with both *v* and *N*. The first type of ordering is easily grasped intuitively, The second is a surprise. *N*-growth is an ordering factor. For N>4 a small two body interaction strength *v* is enough for the system to attain *D* values close to the maximum possible ones. We will call this effect the *N ordering one*. It takes place both in the AFP and in the Lipkin models at moderately high temperatures (Figure 3 and Figure 4). At low *T* the scenario becomes richer. In the AFP case (Figure 5) at low *T* the *N* ordering effect becomes sharper than at high *T*, but with a new ingredient. The effects of the level crossing described above with reference to Figure 2 become also sharper here. In the Lipkin case at low *T* (Figure 6) the *N* effects reverse thhemselves in relation to what happens with the AFP. Here disorder rapidly grows with *N*.

- AFP: order grows with *N*
- Lipkin: Disorder grows with *N*.

Our information theory quantifier *D* is able to show that the two many body systems at hand are quite different ones.

## 7. AFP’s and Lipkin’s Associated Probability Distributions (PDs) versus the Coupling Constant for *N* = 14

**Figure 7:**
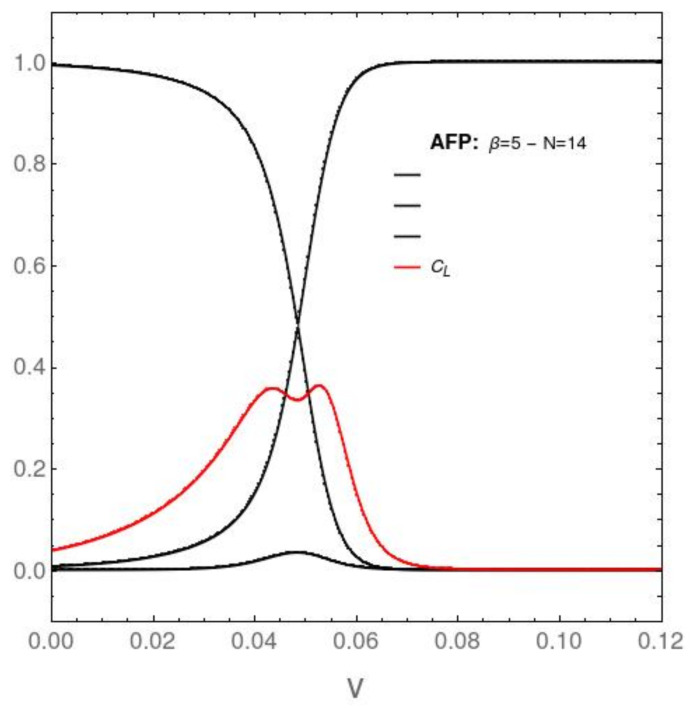
AFP: Three lowest-lying energy eigenvalue’ probabilities $P_{n}$ [vertical axis] (n=0,1,2) versus the interaction coupling constant *v* [horizontal axis] for β=5 and N=14. $P_{0}$ (left-most black curve that reaches unity at v=0) and $P_{1}$ (black curve that reaches unity at large *v*). These two curves cross at v=0.04822, which explains the disequilibium minima of $D_{AFP}$. The associated statistical complexity $C_{L}=S_{AFP}D_{AFP}$ (red curve) displays at the crossing a typical double maximum with a minimum in between, well known in the Literature. $P_{2}$ is very small (bottom black curve). The interesting physics takes place at small values of the pertinent coupling constant.

**Figure 8:**
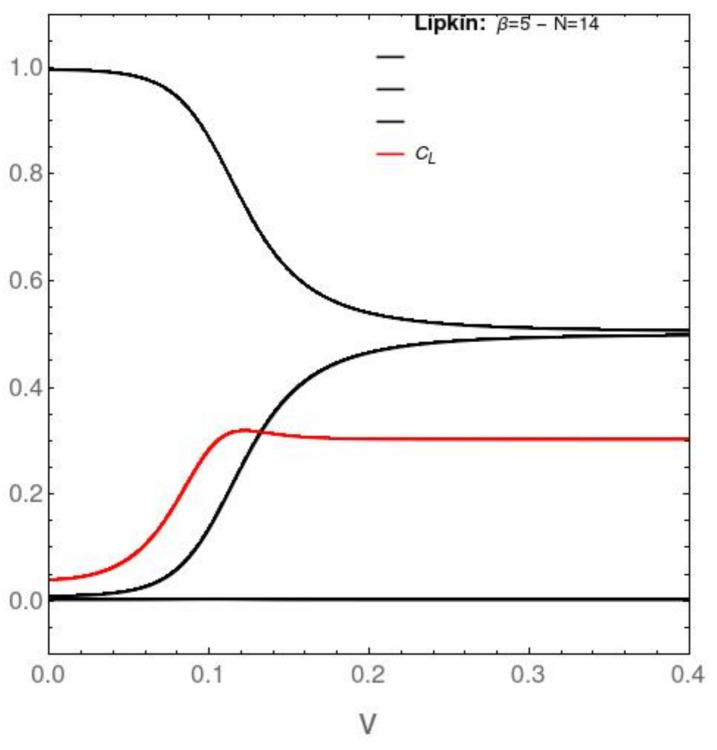
$P_{n}$ [vertical axis] versus the interaction coupling constant *v*. This is the same as above, for $P_{0}$, $P_{1}$, and $P_{2}$, but in the case of the Lipkin model. No probability crossings are detected here. $P_{2}$ is negligible. We find degeneration instead of the two first probabilities. The statistical complexity (red curve) grows as we approach the $P_{0}-P_{1}$ probability degeneracy, and then stabilizes itself. The interesting physics takes place at small values of the pertinent coupling constant.

**Figure 9:**
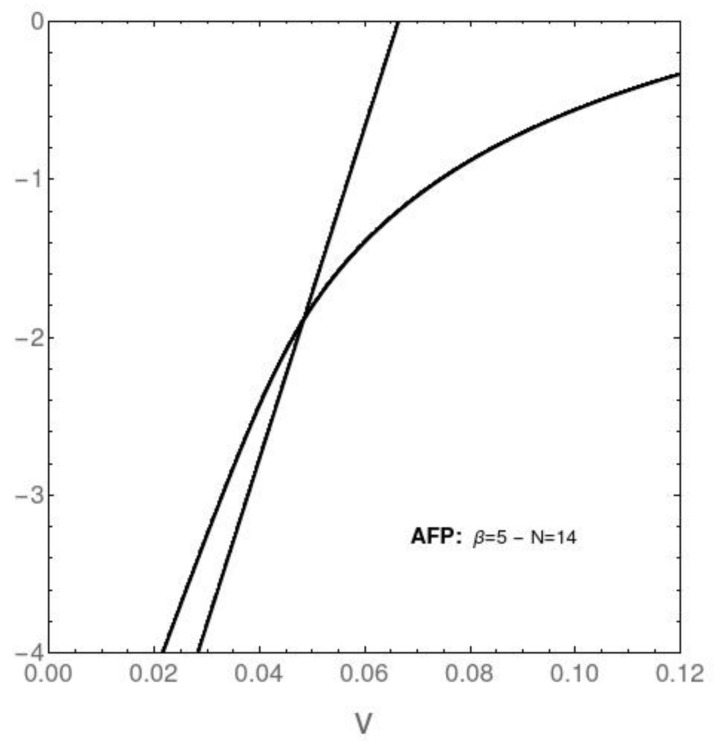
AFP model. Vertical axis: energies pertaining to the two lowest-lying eigenvalues versus [horizontal axis] the coupling constant *v* for N=14. The system’s ground state changes at v∼0.04, where a level-crossing is detected.

**Figure 10:**
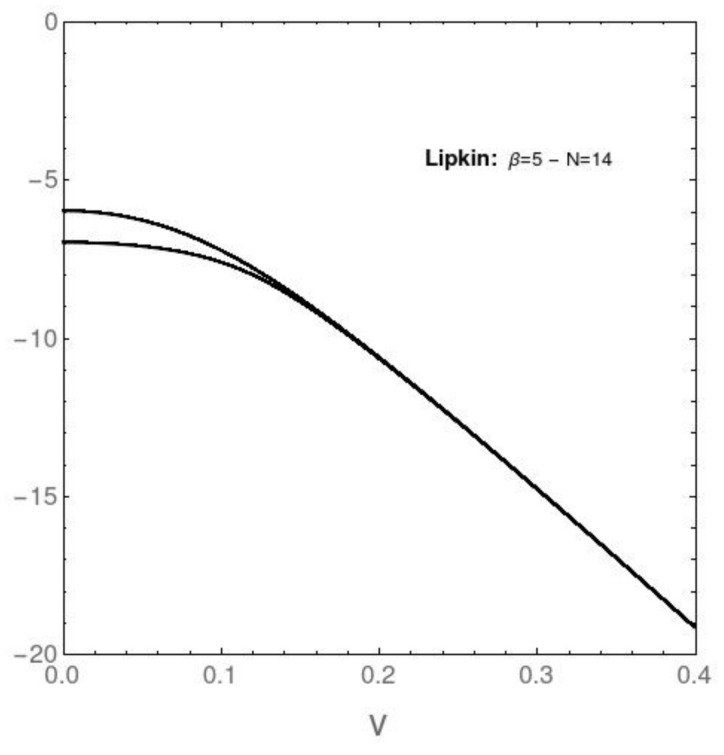
Lipkin model. Vertical axis: energies pertaining to the two lowest-lying eigenvalues versus [horizontal axis] the coupling constant *v* for N=14. No level-crossings are detected, but, instead, degeneration of the two lowest lying levels at small *v*.

It is quite instructive to study to analyze the behavior of $P_{0},\;P_{1},\;\ldots ,\;P_{14}$. We do this in Figure 7, Figure 8, Figure 9 and Figure 10. We will see that the $P_{n}$ are vanishingly small for n>2. All the dynamics is dominated by the behavior of $P_{0}$, $P_{1}$, and $P_{2}$, an a priori unexpected result. We will incorporate in the pertinent graphs the behavior of an, up to this point, not yet employed information-theoretic quantifier called the statistical complexity $C_{L}=SD$ (red curve in Figure 7 and Figure 8). For N=14, in the AFP instance (Figure 7), the minimal $D_{AFP}$ occurs at v=0.048586. The graphs below depict just the probabilities fot n=0,1, and 2, since the remaining ones are extremely small.

Figure 8 deals with the Lipkin model in the same manner as Figure 7 does it with the AFP model.

## 8. The Only Pure Quantum Information (at *T* = 0)

We speak in Figure 9 and Figure 10 about that information referring only to the Hamiltonian’s eigenvalues. No statistics is involved and T=0. As an example, we display below the behavior of the energies pertaining to the two lowest-lying eigenvalues versus the coupling constant’s *v* value for both the AFP (Figure 9) and Likin (Figure 10) instances. On the basis of this scant quantum information, and with the help of statistical quantifiers, we might say that one could have built the edifice described above.

## 9. Conclusions

We have discussed the quantum statistics of two well known exactly soluble finite many fermion systems that the Literature shows to have been extremely useful in shedding light on the intricacies of quantum many fermion physics. Having exact solutions at hand has been very helpful in understanding finite many fermion behavior at also finite temperature.

Indeed, interesting insights have been obtained by appeal to two different but exactly soluble many fermion systems and we have shown how their differences can be best appreciated by recourse to information theoretic tools.

The present analysis indicates that complexity tends to increase when the coupling-constant strengths decrease. A similar trend is observed with regards to the number of fermions in the system: systems with few fermions are endowed with a much larger complexity-degree than systems with a large number of fermions. If these circumstances turn out to be universal features of multi-fermions systems, applicable also to multi-electrons systems, this may have some bearing on the fact that atoms with a relatively small atomic number (roughly in the ”north-east” zone of the periodic zone around C, N, and O) are the ones exhibiting the most varied features and rich chemical properties.

A remarkable benefit of the information-theoretic approach to physics is that it suggests new connections between apparently unrelated problems. For instance, information-theoretic ideas based on Fisher information helped to establish interesting links between Schroedinger’s equation and Boltzmann equation [39]. We hope that. in a similar vein, the present study may stimulate research into the connections between different kinds of many-fermion systems.

We intend to deal with SU(N) symmetries in a future effort.

## Data Availability

Everyting that might be needed is here.
